# Internal Jugular Vein Thrombosis After Microwave Ablation of Cervical Lymph Node Metastasis in Papillary Thyroid Microcarcinoma: A Case Report

**DOI:** 10.3389/fendo.2022.792715

**Published:** 2022-04-27

**Authors:** Ying Liu, Xi-Ju Wang, Jin-Ling Wang, Li-Hong Liu, Shuo-Ran Zhao, Shou-Jun Yu, Bei-Bei Yang, Qing-Ling Xu, Jin-Ke Li, Shu-Rong Wang

**Affiliations:** ^1^Department of Ultrasound Intervention, Yantai Hospital of Shandong Wendeng Orthopaedics and Traumatology, Yantai, China; ^2^Department of Ultrasound, Yantai Affiliated Hospital of Binzhou Medical University, Yantai, China; ^3^Intensive Care Unit, Yantai Affiliated Hospital of Binzhou Medical University, Yantai, China

**Keywords:** internal jugular vein, thrombosis, microwave ablation, lymph node, cervical, papillary thyroid carcinoma

## Abstract

In this study, two patients with papillary thyroid carcinoma and lymph node metastasis were treated by Dr. Shurong Wang’s team and are reported. The two patients refused surgery and underwent microwave ablation (MWA) of the thyroid and lymph node lesions. Ultrasound review 2 days after MWA revealed internal jugular vein thrombosis. Patient #1 received low molecular weight heparin calcium injection, Xueshuantong injection, Xiangdan injection, and rivaroxaban. Patient #2 was treated with enoxaparin sodium injection, Xueshuantong injection, urokinase, and warfarin sodium tablet. The thrombus was successfully managed in each patient using anticoagulant treatment. Such complication of MWA has not been reported in many cases before. According to the relevant literature, thrombosis after thyroid cancer ablation might be related to subclinical hypothyroidism, increased heme oxidase 1 (HO-1) levels in the blood of patients with papillary thyroid cancer, and increased platelet content and mean platelet volume in patients with thyroid cancer. No specific cause of thrombosis was identified in the two cases reported here. No recurrence was observed after 1 (patient #1) and 4 (#2) years of follow-up. In conclusion, patients with papillary thyroid carcinoma and lymph node metastasis should undergo color Doppler ultrasound of the neck after MWA of thyroid lesions and neck metastasis.

## Introduction

Thyroid cancer is the most common malignant tumor of the endocrine system (1). Papillary thyroid carcinoma is the most common type of thyroid cancer, with a high frequency of cervical lymph node metastasis but a slow course and low mortality (2). There were 60,220 cases of thyroid cancer in the United States in 2013 and 90,000 new cases of thyroid cancer in China in 2015 (1). Due to the good biological behavior and slow growth of papillary thyroid microcarcinoma, some patients carry lesions all their lives without disease progression. Therefore, some authors believe that some small cancers can be observed without any treatment. For example, Ito et al. (3) reported the observation results of 340 patients with papillary thyroid microcarcinoma operated on or not; after an average follow-up time of 74 months, no significant differences were found in lymph node metastasis and prognosis between the two groups. Another study by Ito et al. (4) followed 1235 cases of thyroid microcarcinoma for 6 years; lesions increased in 58 cases (4.6%), and lymph node metastasis occurred in 19 cases (1.5%).

Minimally invasive thyroid treatment technologies are available for patients with microcarcinoma who refuse thyroidectomy for fear of postoperative pain or cosmetic reasons. Ultrasound-guided microwave ablation (MWA) is widely used in the alternative treatment of primary and metastatic liver cancer, lung, kidney, and adrenal malignancies (5). In addition, MWA in treating benign thyroid neoplasms has achieved considerable efficacy (6).

This paper reports two patients with papillary thyroid carcinoma and lymph node metastasis. Both patients developed internal jugular vein thrombosis after the MWA of thyroid carcinoma and lymph node metastases. Such complication of MWA has not been reported in many cases before.

## Case Presentation

### Case 1

**Figure 1:**
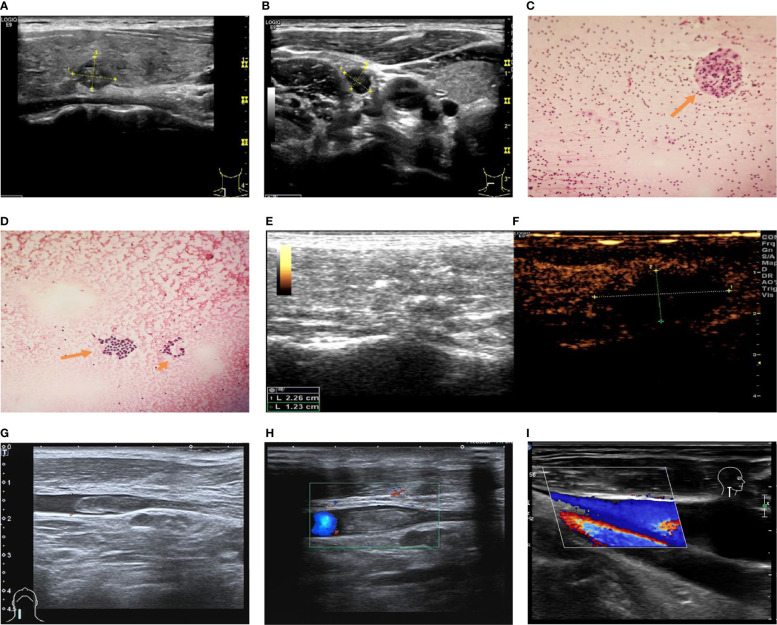
Ultrasound and flow chart of Patient #1 after ablation. **(A)** Preoperative ultrasound of thyroid nodule. **(B)** Preoperative ultrasound of lymph node. **(C)** Thyroid FNA smear: epithelial cells with dysplasia (arrow). **(D)** Cervical lymph node FNA smear: lymphocytes and epithelial cells with dysplasia (arrow). **(E, F)** Ultrasound after extended ablation of papillary thyroid carcinoma of the right lobe of thyroid of the patient immediately after postoperative (local no contrast medium filling). **(G)** Two-dimensional ultrasonography of internal jugular vein thrombosis on the second day after ablation. **(H)** Thrombus flow diagram of the right internal jugular vein on the second day after ablation. **(I)** One month after thrombolysis, venous thrombosis disappeared, and venous blood flow returned to normal.

A 28-year-old female patient was admitted to the Oncology Department of Yantai Affiliated Hospital affiliated to Binzhou Medical College because thyroid nodules were found during a physical examination on September 20, 2019. She had no family history of thyroid diseases. Blood cell analysis revealed red blood cells (RBC) at 4.79×10^12^/L, hemoglobin (HGB) at 83 g/L ↓, hematocrit (Hct) at 29.1% ↓, mean corpuscular volume (MCV) at 60.8 fL ↓, mean corpuscular hemoglobin (MCH) at 17.3 pg ↓, average hemoglobin concentration (MCHC) at 285 g/L ↓, platelets (PLT) at 256×10^9^/L, and mean platelet volume (MPV) at 7.0 fL. For thyroid function, free triiodothyronine (FT3) was 4.44 pmol/L, free thyroxine (FT4) was 13.50 pmol/L, thyroid-stimulating hormone (TSH) was 10.00 µIU/mL ↑, thyroglobulin antibody (TGAb) was 260.20 U/mL ↑, and thyroid peroxidase antibody (TPOAb) was >1300 U/mL ↑. Carbohydrate antigen 125 (CA125) was 35.6 U/mL ↑. For coagulation, plasma fibrinogen (Fib) was 1.59 g/L ↓, and D-dimer was 1.24 µg/mL ↑. Other tests were unremarkable. Laryngoscopy and chest radiography were negative. On ultrasound, a hypoechoic nodule of 1.10×0.75×0.63 cm could be seen in the right lobe of the thyroid gland, with irregular shape and unclear boundary (**Figure 1A**). Strong spot-like echoes could be seen inside. Hypoechoic nodules were seen in levels IV and VI of the right neck. The larger one was located at the right, with a size of about 1.32×0.63 cm, irregular in shape, an unclear boundary between the dermis and medulla, without hilum (**Figure 1B**). Biopsy of the thyroid nodule revealed papillary thyroid carcinoma (**Figure 1C**). Fine-needle aspiration (FNA) of the levels IV and VI lymph nodes revealed lymphocytes and epithelial cells with dysplasia, consistent with lymph node metastasis of papillary thyroid carcinoma (**Figure 1D**).

According to the ultrasound classification of thyroid nodules and cytology results, the patient was diagnosed with papillary thyroid carcinoma and cervical lymph node metastasis. Surgical treatment was recommended, but the patient was young and suffered from anemia, and she refused surgery. The physician strongly recommended ultrasound-guided MWA, which Dr. Shurong Wang performed on September 25, 2019. Expanded ablation was performed for papillary thyroid carcinoma of the right lobe of the thyroid (with a maximum ablation range of 2.26 × 1.23 cm) and four cervical lymph nodes. Postoperative contrast-enhanced ultrasound showed no enhancement of the thyroid nodules after ablation (**Figures 1E, F**). The thyroid ultrasound on the second day after ablation showed necrotic changes in the right lobe of the thyroid and right cervical lymph nodes. Heterogeneous hypoechogenicity was observed in the middle and lower segment of the right internal jugular vein, with a length of about 3.56 cm and a width of about 0.50 cm (**Figure 1G**). A small number of blood flow signals were observed around the hypoechogenic signals (**Figure 1H**). After thrombolytic therapy (low molecular weight heparin calcium injection 4100 IU, q 12 h, subcutaneous injection, 5 days; Xueshuantong injection 400 mg added into 0.9% sodium chloride injection 250 ml, qd, IVGTT, for 5 days; Xiangdan injection 20 ml added into 5% glucose injection 250 ml, qd, IVGTT, for 5 days; rivaroxaban 15 mg, bid, po, for 7 days), the venous thrombosis disappeared, and venous blood flow had returned to normal at the 1-month reexamination (**Figure 1I**). The patient was kept on suppressive treatment after ablation. No recurrent lesions were found in the cervical lymph nodes and thyroid during 1 year of postoperative follow-up.

### Case 2

**Figure 2:**
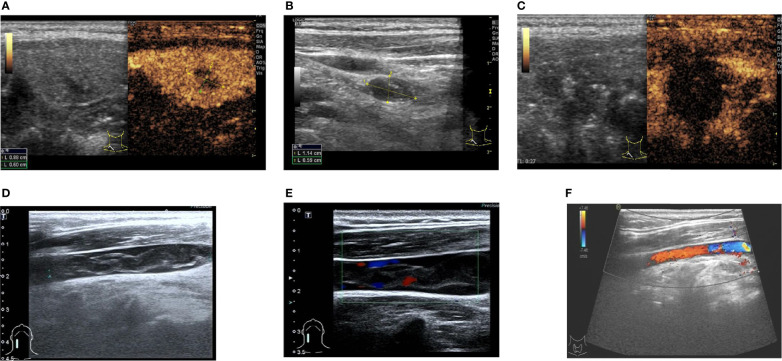
Ultrasound and flow chart of Patient #2 after ablation. **(A)** Preoperative ultrasound of thyroid nodule. **(B)** Preoperative ultrasound of lymph node. **(C)** Ultrasound after extended ablation of papillary thyroid carcinoma of the right lobe of thyroid of the patient immediately after postoperative (local no contrast medium filling). **(D)** Two-dimensional ultrasonography of internal jugular vein thrombosis on the second day after ablation. **(E)** Thrombus flow diagram of the right internal jugular vein on the second day after ablation. **(F)** One week after thrombolysis, venous thrombosis disappeared, and venous blood flow returned to normal.

A 49-year-old female patient was admitted to the Department of Oncology of Yantai Affiliated Hospital of Binzhou Medical College because thyroid nodules were found during a physical examination on January 1, 2016. She had no family history of thyroid disease. The blood cell analysis showed a neutrophil ratio (Neut%) at 36.9% ↓, lymphocyte ratio (Lymph%) at 48.3% ↑, RBC at 4.20×10^12^/L, HGB at 128 g/L, Hct at 37.2%, MCV at 88.6 fL, MCH at 30.4 pg, MCHC at 343 g/L, PLT at 196×10^9^/L, and MPV at 10.1 fL. FT3 was 5.20 pmol/L, FT4 was 14.83 pmol/L, TSH was 2.28 µIU/mL, TGA was 38.90 U/mL, TPOAb was 53.80 U/mL, and CA125 was 8.70 U/mL. Preoperative laryngoscopy and chest radiography were negative. Ultrasound of the thyroid showed a hypoechoic nodule of 0.88×0.72×0.76 cm in the right lobe of the thyroid, with an irregular shape, unclear boundary, uneven internal echo, and spot-like strong echo (**Figure 2A**). Blood flow signals could be seen around and inside the nodule. Two lymph node lesions could be detected in level IV of the right neck; the larger was about 1.14×0.59 cm, with a regular shape, clear boundary, and uneven internal echo (**Figure 2B**). Contrast-enhanced ultrasound showed uneven and low enhancement of the nodules in the right lobe of the thyroid gland. Cytology results revealed papillary thyroid carcinoma with right cervical lymph node metastasis, galectin-3 (+), Ki67 (+), MC (+), CD56 (-), and CK19 (+).

Surgical treatment was recommended, but the patient refused the operation (for fear of pain and esthetic reasons) and insisted on MWA. The patient underwent ablation by Dr. Shurong Wang on January 5, 2016. Expanded ablation was performed for the papillary thyroid carcinoma of the right lobe of the thyroid and two cervical lymph nodes. Postoperative contrast-enhanced ultrasound showed no enhancement of thyroid nodules after ablation (**Figure 2C**). Heterogeneous hypoechogenicity was observed in the middle and lower segment of the right internal jugular vein, with a length of about 3.56 cm and a width of about 0.50 cm according to 2D ultrasound and color Doppler ultrasound (**Figures 2D, E**). Thrombolytic therapy was given (enoxaparin sodium injection 6000 IU, q 12 h, subcutaneous injection, 1 week; 0.5 g Xueshuantong added into 250 ml of normal saline, qd, and IVGTT, for 5 days; urokinase 200,000 IU added into 0.9% normal saline, bid and IVGTT, for 5 days; warfarin sodium tablet 2.5 mg, qd, po, started on the sixth day of medication, for one week). After 1 week, the thrombus disappeared, and venous flow was restored (**Figure 2F**). The patient was kept on suppressive treatment for 3 years after ablation. No recurrent lesions were found in the cervical lymph nodes and thyroid gland during the postoperative follow-up of >4 years

## Discussion

There are few reports of internal jugular vein thrombosis after MWA of thyroid lesions. The cervical lymph node metastasis was located behind the internal jugular vein in the two cases reported here. During the ablation of lymph nodes, normal saline should be injected between the lymph node and the internal vein to protect the internal jugular vein from heat damage. The blood flow of the ipsilateral internal jugular vein was observed to be normal immediately after the operation. Routine local compression and cold compress were applied for 60 min after the operation. The internal jugular vein thrombosis in the two patients might be related to compression caused by edema and the cold compress. Still, many patients did not develop internal jugular vein thrombosis under the same conditions. In the past 5 years, the authors treated 54 patients with cervical lymph node metastasis of thyroid cancer with MWA. Thirty-eight of them (70.4%) were located in level IV of the neck. Each patient underwent postoperative cervical vascular ultrasound. The ablation needle models KY-2000 and KY-2450A-1 were used. The ablation power of MWA was 30 W in both patients. The ablation range was expanded by 0.5 cm around the thyroid cancer. The cervical lymph nodes underwent conformal ablation. The distance from the internal jugular vein was about 1 cm. In addition, sufficient spacer fluid was injected around the lymph nodes during the ablation to prevent heat damage. Therefore, the possibility of intraoperative jugular vein injury is very small.

According to the literature, internal jugular vein thrombosis after MWA might be related to the following factors. First, Erem et al. (7) reported that certain coagulation factors might be increased in the blood of patients with subclinical hypothyroidism (SH) (7). The main diagnostic indicator of SH is the elevation of TSH in blood, and studies showed that serum TSH levels are independently associated with thrombosis (8). TSH is related to the change of gene expression of endothelial cells, and the increase of TSH might influence the expression of the endothelial nitric oxide synthase (PGI2) gene and induce endothelial cell dysfunction and thrombosis (9). Patient #1 had SH, which might be related to venous thrombosis after MWA. Second, Chadarevian et al. (10) reported that patients with mild hypothyroidism had decreased DD levels, increased plasma Fib, and decreased fibrin degradation, which could easily lead to local thrombosis. In this study, Patient #1 showed a decrease in Fib and an increase in DD. Other coagulation items were all within the normal range. On the other hand, Patient #2 showed normal coagulation parameters. Therefore, it was inconsistent with the cases reported here. In addition, only patient #1 had SH. Third, elevated levels of heme oxidase 1 (HO-1) have been observed in the blood of patients with papillary thyroid carcinoma (11). During the process of heme catabolism, HO-1 promotes the production of carbon monoxide (12). Increased plasma carbon monoxide concentrations promote coagulation by adhesion to fibrinogen (13), which leads to thrombosis. Both patients in this study developed venous thrombosis after MWA of papillary thyroid carcinoma, which might cause an enhancement of the coagulation response induced by a series of physiological reactions of cancer cells or through some stimulation. Fourth, increased platelet content and mean platelet volume in patients with thyroid cancer could induce thrombosis (14). Both patients in this article were in the normal range. Fifth, thyroid cancer itself might be prone to thrombosis, and the side effects of levothyroxine inhibitory might increase VTE risk. Endothelial dysfunction, primary or secondary blood coagulation, and fibrinolysis pathway disorders can enhance the blood coagulation response (15, 16).

In this report, right internal jugular vein thrombosis occurred in two patients after MWA of papillary thyroid carcinoma neck metastases. It might be caused by impaired endothelial function after ablation. In this study, the common characteristics of the two cases were thyroid cancer and lymph node metastasis in level IV of the right neck, which was treated by MWA. The right cervical lymph nodes are located around the right internal jugular vein. Cryogenic isolating fluid was injected during the ablation process to compress the internal jugular vein. After the surgery, an ice bag was used to apply pressure on the neck, inducing thrombosis. Still, the exact causes remain to be determined.

## Data Availability Statement

The original contributions presented in the study are included in the article/supplementary material. Further inquiries can be directed to the corresponding author.

## Ethics Statement 

The studies involving human participants were reviewed and approved by [Ethics Committee of Yantai affiliated hospital of Binzhou Medical University] [Approval number: F-KY-0059-20151001001-01]. The patients/participants provided their written informed consent to participate in this study.

## Author Contributions

S-RW and YL contributed to the conception and design of the study. X-JW, J-LW, L-HL, S-JY, and B-BY organized the database. S-RZ performed the statistical analysis. YL wrote the first draft of the manuscript. S-RW, Q-LX, and J-KL wrote sections of the manuscript. All authors contributed to the article and approved the submitted version.

## Funding

The authors declare that this study received funding from the Shandong Natural Science Foundation Program, Molecular immune mechanism of ultrasound-guided microwave implant ablation for thyroid cancer [Grant number: ZR2017LH054]. The funder had the following involvement with the study: study design, interpretation of data, the writing of this article, and the decision to submit it for publication.

## Conflict of Interest

The authors declare that the research was conducted in the absence of any commercial or financial relationships that could be construed as a potential conflict of interest.

## Publisher’s Note

All claims expressed in this article are solely those of the authors and do not necessarily represent those of their affiliated organizations, or those of the publisher, the editors and the reviewers. Any product that may be evaluated in this article, or claim that may be made by its manufacturer, is not guaranteed or endorsed by the publisher.
